# Structured physical exercise for bipolar depression: an open-label, proof-of concept study

**DOI:** 10.1186/s40345-023-00294-8

**Published:** 2023-04-21

**Authors:** Beny Lafer, Cicera Claudinea Duarte, Julia Maria D’Andrea Greve, Paulo Roberto dos Santos Silva, Karla Mathias de Almeida, Gabriel Okawa Belizario, Lucas Melo Neves

**Affiliations:** 1 Bipolar Disorder Program (PROMAN), Department of Psychiatry, University of São Paulo Medical School, Rua Dr. Ovídio Pires de Campos, 785, São Paulo, Brazil; 2 Movement Studies Laboratory, Department of Orthopedics and Traumatology, University of São Paulo Medical School, São Paulo, Brazil; 3 Post-graduate Program in Health Sciences, Santo Amaro University, São Paulo, Brazil

**Keywords:** Physical activity, Physical exercise, Mental health, Bipolar disorder, Depression

## Abstract

**Background:**

Physical exercise (PE) is a recommended lifestyle intervention for different mental disorders and has shown specific positive therapeutic effects in unipolar depressive disorder. Considering the similar symptomatology of the depressive phase in patients with bipolar disorder (BD) and unipolar depressive disorder, it is reasonable to suggest that PE may also be beneficial for bipolar depression. However, there is an absence of studies evaluating the antidepressant effect of a structured PE intervention in BD.

**Methods:**

This is an open-label, single-arm study trial. Fifteen patients with a diagnosis of BD Type I or Type II, presenting a depressive episode were included in the study. After physical and functional evaluation, patients participated in supervised training sessions with aerobics followed by strength exercises, three times per week, for 12 weeks (36 training sessions). Depressive and manic symptoms were assessed at baseline and 2, 4, 8, and 12 weeks. Additionally, quality of Life and functioning were assessed at baseline and 4, 8, and 12 weeks). Finally, we tested cardiorespiratory fitness, muscle strength and body composition at baseline and week-12.

**Results:**

The mean (± SD) *Montgomery Asberg Depression Rating Scale* (MADRS) score at baseline was 23.6 ± 8.3 points and after 12 weeks of PE the mean score was 10.2 ± 4.8 points. Nine patients (82%) presented an antidepressant response defined as a reduction of more than 50% of depressive symptoms at week 12 with five of those patients (45%) presenting criteria for full remission. A large and significant Cohen’s D Effect Size (pre-post) was verified for MADRS reduction [1.98 (95% Confidence interval = 0.88 to 3.08)]. We did not detect a significant change in manic symptoms, functioning, and quality of life during the 12-week follow-up. At week-12, all patients increased their muscular strength (one repetition maximal test − 1RM) and reduced the percentage of body fat (spectral bioelectrical impedance analysis).

**Conclusions:**

This study, using rigorous criteria and a structured intervention, provides valid pilot data, showing the feasibility of a structured PE intervention for the treatment of depressive symptoms in BD, and suggesting a potential adjunctive antidepressant effect. Moreover, PE showed a positive impact on muscle strength and body composition. This should be further verified by randomized controlled studies.

## Background

Bipolar disorder (BD) affects more than 100 million people worldwide and is associated with high disability and premature mortality (Ferrari et al. 2022). The treatment (pharmacological and psychosocial) is recommended soon after disease onset (Carvalho et al. 2020), and recent evidence has emerged. Lifestyle interventions may present benefits as a complementary therapeutic strategy (Firth et al. 2020). Physical exercise (PE) is an important lifestyle intervention for different mental disorders and convergent evidence indicates its role in primary prevention and symptomatic improvement across a spectrum of mental disorders, especially in anxiety and depressive disorders (Firth et al. 2020).

In patients with major depressive disorder (MDD), several studies have shown an antidepressant effect of PE (Heissel et al. 2023). Given the similarities between depressive periods in patients with BD and MDD, it is reasonable to speculate that the same benefits of PE may apply to bipolar depression. However, there is an evident disparity between the studies on MDD versus BD (Lafer et al. 2022). Confirming the scarcity of data in BD, a recent meta-review of PE and psychiatric disorders (27 systematic reviews and 16 meta-analyses, a total of 152 randomized clinical trials) identified four meta-analyses (90 randomized clinical trials) with PE in MDD, but no randomized clinical trials in BD (Ashdown-Franks et al. 2020). Moreover, PE is a potentially modifiable behavior and a good indicator and independent predictors of the physical and mental health status of patients with BD (Ashton et al. 2020; De Sá Filho et al., 2016; Firth et al. 2020; Melo et al. 2016; Miranda-Pettersen et al. 2020; Sun et al. 2020).

In view of the potential physical advantages of PE, we highlight the World Health Organization Recommendations (Bull et al. 2020), which emphasize the necessity of engaging in two types of PE, aerobic (e.g. walking or running) and strength training (e.g. body muscles to move or try to move against an opposite force regularly). In fact, adult guidelines include strong recommendations on weekly volumes of aerobic and strength training for health (Bull et al. 2020). Importantly, the combination of aerobic and strength training in the same section can promote gains both in cardiorespiratory fitness (peak oxygen consumption) and muscular strength (Pito et al. 2021). Finally, peak oxygen consumption (Han et al. 2022) and muscular strength (Jochem et al. 2019) is inversely related to mortality risk in a variety of acute and chronic conditions, therefore, increasing cardiorespiratory fitness and muscular strength is especially important for BD patients, given its reported and replicated lower life expectancy (Chan et al. 2022).

In recent studies with adolescents with BD a link between cardiorespiratory fitness and depressive symptoms was identified (Popel et al. 2021). Intriguingly, behavior change counselling was shown to be effective in encouraging adolescents with BD to engage in more aerobic training (Khoubaeva et al. 2023), but there is a scarcity of research trials on adults with BD and exercise training. In fact, only a small number of pilot studies with adults with BD was conducted (Ng et al. 2007; Sylvia et al. 2013b, 2019) but none of the studies utilized structured PE program to assess its role as an add-on therapy for bipolar depression. In addition to previous limitations, the possible negative effect of PE in BD needs more investigation. Wright et al. through semi-structured interviews investigated the perception of BD patients about the relationship between PE and BD (Wright et al. 2012). The authors identify narratives about exercise practices as a potential trigger for manic episodes. Based on these interviews, the authors call attention to PE can be a “double-edged sword” for BD, suggesting an antidepressant effect of PE, but a possible effect to induce manic episodes.

PE reduces depressive symptoms and increases cardiorespiratory fitness. Moreover, it is worth investigating its impact on other variables such as muscular strength and body composition, especially due to the high rates of obesity and sedentarism encountered in BD (Firth et al. 2020). Finally, the evaluation of the impact of exercise in manic symptoms, quality of life and functioning may show other potential benefits as well as possible side effects of PE in BD.

Therefore, this open-label, single-arm pilot study aimed to assess the potential antidepressant benefits as an augmentation strategy in patients with BD utilizing a structured protocol of PE (supervised aerobic plus strength training) as well as assessing adherence and tolerability of the intervention for 12 weeks. Furthermore, we also aimed to evaluate the potential impact of our exercise protocol on cardiorespiratory fitness, muscular strength and body composition.

## Methods

### Participants

This open trial followed the guidelines of the Consolidated Standards of Reporting Trials (CONSORT) 2010 statement: extension to pilot and feasibility trials (Eldridge et al. 2016). The recruited patients were outpatients at the Bipolar Disorder Program (PROMAN) at the Institute of Psychiatry of the *Hospital das Clínicas* of the University of Sao Paulo Medical School. All patients were interviewed for the study after providing informed consent. Eligibility criteria included the following: (a) Diagnosis of BD Type I or Type II by Structured Clinical Interview for DSM (SCID-P); (b) Aged between 18 and 55 years; (c) Mild or moderate depressive episode at recruitment - Score on Montgomery–Åsberg Depression Rating Scale (MADRS) ≥ 14 points; (d) No regular PE in the previous six months; (e) Using lithium, quetiapine, valproate, lamotrigine, or a combination of these stabilizers for at least four weeks, without remission of the depressive episode; (f) Patients who were on antidepressants were required to have been taking a stable dose for at least four weeks; (g) No medication change in the four weeks prior to study entry; (h) Patients using a maximum of 2 mg of Lorazepam and/or 10 mg of Zolpidem for insomnia; (i) Body mass index (BMI) ≤ 40. The exclusion criteria were as follows: (a) Being in a manic or hypomanic episode at recruitment - Score on the Young Mania Rating Scale (YMRS) > 12 points; (b) having an organic mental disorder; (c) Abuse of alcohol or illicit drugs in the previous two weeks; (d) Using of beta-blocker.

### Study design and procedures

This study was a prospective, single-center, open-label, single-arm pilot trial conducted from December 2016 to March 2020. As this is a pilot study, no sample size calculations were performed. Before enrollment, informed consent was obtained from all patients and the local ethics committee approved the study (protocol number 3.119.424). This study was conducted by the Declaration of Helsinki (Goodyear et al. 2007). Individualized and supervised training sessions of aerobic plus strength training (added to pharmacological treatment), were performed three times a week (Monday, Wednesday, and Friday) for 12 weeks (36 training sessions).

All patients were diagnosed with BD type I or II based on the Structured Clinical Interview for DSM IV (SCID-P) (First and Gibbon 2004) and followed in the outpatient service for at least one year before the beginning of this study. In addition, symptoms of depression (MADRS) and mania (YMRS) were assessed at baseline and after 2, 4, 8, and 12 weeks. In relation to MADRS scores, we followed the categories of depression severity in patients with BD (mildly depressed - until 18 points, moderately depressed – 19–23 points, markedly depressed – 24–36 points, severely depressed – 37–39 points, and, extremely depression ≥ 40 points) defined by Thase et al. (Thase et al. 2021). Finally, Functioning (*Functioning Assessment Short Test* - FAST) and Quality of Life (*World Health Organization Quality of Life* - WHOQOL-bref) were assessed at baseline and after 4, 8, and 12 weeks. The MADRS and YRMS scales are scales typically used in interventions in patients with BD (assessing symptoms of depression and mania) (Montgomery and Asberg 1979; Young et al. 1978). The FAST is an instrument designed to measure functioning disability and validated in patients with BD (Rosa et al. 2013). The WHOQOL-bref is an instrument developed to assess Quality of Life (Whoqol-Group, 1998).

At baseline, patients were evaluated by a clinical physician in order to assess musculoskeletal disorders, cardiovascular disorders and measure vital signs. If necessary, supplementary laboratory testing was ordered prior to approving the patient to participate in the physical exercise program. The physical fitness test (cardiorespiratory fitness, muscular strength and body composition) was assessed at baseline and after 12 weeks.

### Physical fitness test – cardiorespiratory fitness, muscular strength and body composition

Ergospirometry test mensured cardiorespiratory fitness (peak oxygen uptake - VO_2peak_) used a treadmill (H/P/Cosmos Sports & Medical, Nussdorf-Traunstein, Germany), and was conduced modified Heck protocol (Heck et al. 1985; Santos-Silva et al. 2007), ramp style, with fixed speed and 2% incline increments every minute. The total exercise time test lasted between 8 and 15 min, as previously described in the literature (Buchfuhrer et al. 1983). The cardiac response to the incremental was used as parameter of cardiac health (electrocardiographic signal was recorded throughout the test).

Muscular strength, was measured by the one repetition maximal test (1RM). In resume, a unilateral leg press 45° 1RM testing were performed considering the American Society of Exercise Physiologists’ recommendations (Brown and Weir 2001). The participants ran for 5 min (min) on a treadmill, followed by two warm-up sets. In the first set, the participants performed eight repetitions with an intensity of 50% of their estimated 1RM obtained during the familiarization sessions. In the second set, they performed three repetitions with 70% of their estimated 1RM. A 3-min rest interval was afforded between warm-up sets. After completing the second set, participants rested for 3 min and then had five attempts to achieve their 1RM. Again, a 3-min rest interval was afforded between attempts (Brown and Weir 2001). Finally, percentage of body fat and body muscle mass was measured using a spectral bioelectrical impedance analysis (InBody 270, Biospace Seoul, Korea). In resume, the subjects were instructed to fast for at least three hours, not to perform physical activity the day before the exam, and not to drink alcoholic beverages, coffee, tea, chocolate, and soft drinks.

### Program of exercise

The following exercises were completed under supervision: Aerobic training (treadmill); strength training (specific equipment).

### Aerobic training

Aerobic training on a treadmill (H/P/Cosmos Sports and Medical, Nussdorf-Traunstein, Germany) consisted in 5 min at 65–70% of heart rate maximum (HR_max)_, 20 min at 70–85% of HR_max_, and 5 min at 65–70% of HR_max_.

The intensity was based on a percentage of the HR_max_, which was checked in ergospirometry test. All patients used in all section of training a heart rate monitor (Polar H7, Polar Electro Oy, Kempele, Finland) with Polar Beat.

### Strength training

The protocol consisted of five exercises [Leg press, Leg extension, Seated crunch machine (abdominal), Bench press, and Rower machine], with specific equipment (Multiflex, Biodelta®, Brazil), been realized by three sets of 15 repetitions with one to two minutes intervals.

The strength training during the first two weeks, self-selected loads were used due to the need to learn the movement. After this period, loads of 15 repetitions were used, and in each training session, if the subject was able to perform more than 15 repetitions, the load was adjusted.

### Statistical procedures

The data were analyzed using IBM SPSS Statistics, version 22 (SPSS Inc., Chicago, IL, USA). Data are described as individual values. The mean scores (+- SD – standard deviation) of eleven patients and groups are presented. The Cohen’s D Effect Size (Cohen 2013) – [Cohen’s d = (M2 - M1) ⁄ SD pooled] [SD pooled = √ ((SD1 + SD2) ⁄ 2)] with 95% confidence interval (CI) was used to determine the magnitude of the difference detected between baseline and 12 weeks. The interpretation of effect size (ES) used was: 0 to < 0.30 | small, |> 0.30 | to | <0.8 | medium, and |> 0.80 | large. As this is a feasibility study, a formal sample size calculation was not performed.

## Results

**Fig. 1:**
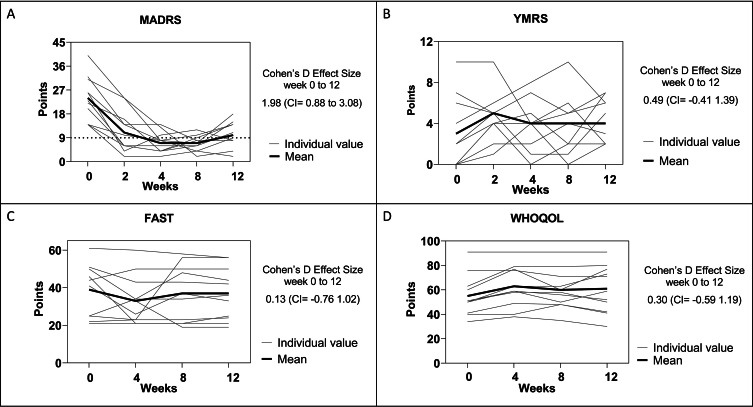
Results for depressive symptoms, manic symptoms, functioning and quality of life (Individual and mean value). Panel A = MADRS (Montgomery-Asberg Depression Scale); Panel B = YMRS (Young Mania Rating Scale); Panel C = FAST (Functioning Assessment Short Test); Panel D = WHOQOL (World Health Organization Quality of Life). 9-point line in panel A = remission. Change between baseline and 2, 4, 8, and 12 weeks and the Cohen’s d effect size

The mean (± SD) Montgomery Asberg Depression Rating Scale (MADRS) score at baseline was 23.6 ± 8.3 points and after 12 weeks of physical exercise the mean score was 10.2 ± 4.8 points. Nine patients (82%) presented an antidepressant response defined as a reduction of more than 50% of depressive symptoms at week 12 with five of those patients (45%) presenting criteria for full remission. Moreover, all five patients with a MADRS score higher than 24 (markedly ill or extremely ill), presented at least a 50% reduction in MADRS score at week-12. A large and significant Cohen’s D Effect Size (pre-post) was verified for MADRS reduction [1.98 (95% Confidence interval = 0.88 to 3.08)]. We did not detect a significant change in manic symptoms, functioning, and quality of life during the 12-week follow-up (Fig. 1).

Flowchart details the recruitment process and study inclusion/exclusion. Of the 27 patients who participated in the recruitment, 15 began the intervention, and 11 completed all assessments In relation to subjects who were screened but did participate in the study, three did not meet inclusion criteria and nine declined to participate. Regarding the patients who started but did not complete the intervention, the reasons for not finishing were: One patient was divorced at week-5, one patient had elective surgery at week-2, and two patients had a lack of interest in continuing the activity, one at week-4 and another at week-7.

Of the 11 completers, the mean exercise sessions were 33/36 (92%) showing very good adherence to the protocol.

**Flowchart:**
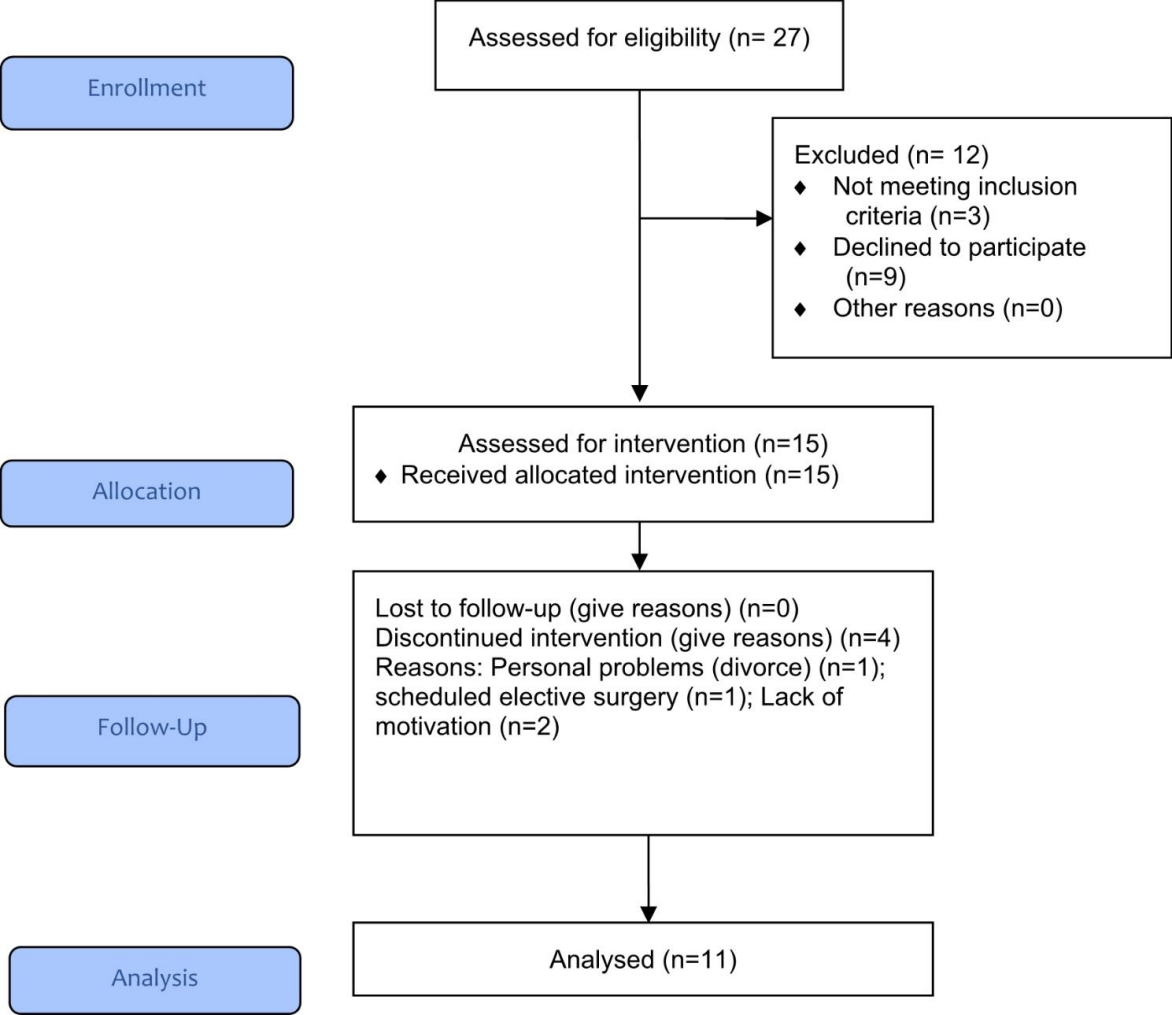
Recruitment process and study inclusion/exclusion

Considering the adverse events observed during the intervention, we highlight the request to reduce exercise load in the early training sessions due to complaints of musculoskeletal pain (6 patients). Moreover, no manic switch was observed during the 12-week follow-up period with none of the patients presenting a score in the YMRS higher than 10 at any point during the study (see Fig. 1).

Table 1 details the main sample characteristics of the participants. The patients were predominantly females, unemployed, and bipolar type 1. Of eleven patients, nine patients had more than BMI ≥ 25 kg/m^2^ (overweight or obese). Prior to the exercise intervention, subjects received four medications on average to treat BD. In relation to MADRS scores, we followed the categories of depression severity in patients with BD defined by Thase et al. (Thase et al. 2021). According to this, three patients were mildly depressed, three patients moderately depressed, four patients markedly depressed and one patient extremely depressed.

**Table 1:** Demographic, clinical characteristics, and pharmacological treatment at baseline (n = 11)

Characteristics of sample (Baseline)	
Female (n)	8
Age (years) – mean ± SD (range)	42 ± 9 (29–55)
Body weight (kg) – mean ± SD (range)	72 ± 14 (51–97)
BMI (kg/m^2^) – mean ± SD (range)	26 ± 4 (21–32)
MADRS Score (points) – mean ± SD (range)	23.6 ± 8.3 (14–40)
YMRS Score (points) – mean ± SD (range)	2.7 ± 3.3 (0–10)
VO2_peak_ (mL/kg/min^− 1^) – mean ± SD (range)	25 ± 7 (16–43)
Muscle strength (kg) – mean ± SD (range)	42 ± 10 (25–55)
Body fat percentage (%) – mean ± SD (range)	31 ± 8 (14–44)
Employed or student (n)	4
Unemployed (n)	7
Married (n)	3
Smoking (n)	1
Bipolar Disorder Subtype and polarity of first episode	
Type 1 (n)	6
Type 2 (n)	5
Polarity of first episode – Depression (n)	10
Polarity of First episode – Mania (n)	1
Stable pharmacotherapies at baseline (by class)	
Antidepressants	
Any antidepressant agent (n)	5
Mood stabilizers and atypical antipsychotics	
Lithium (n)	5
Lamotrigine (n)	2
Quetiapine (n)	6
Olanzapine (n)	1
Carbamazepine (n)	1
Other treatments	
Benzodiazepines (n)	6
Topiramate (n)	3
Haloperidol (n)	1

Note: n = number of patients, BMI = body mass index, MADRS = Montgomery–Åsberg Depression Rating Scale, YMRS = Young Mania Rating Scale, VO_2peak_ = cardiorespiratory fitness, 1RM = one repetition maximum test = muscle strength

Figure 1 presents the MADRS (Panel A) and YMRS (Panel B) data considering baseline and 2, 4, 8, and 12 weeks, as well as the FAST (Panel C) and WHOQOL (Panel D) considering baseline and 4, 8, and 12 weeks. The Graphs show the individual values and mean of the group, and the chart shows the Cohen’s d ES and CI between baseline and 12 weeks for the questionnaires (MADRS, YMRS, FAST, and WHOQOL).

In relation to change in MADRS score domains, we observed (baseline and 12 weeks): (I) Apparent sadness = 2.5 to 1.2 points; (II) Reported sadness = 4.0 to 0.8 points; (III) Inner tension = 2.9 to 1.3 points; (IV) Reduced sleep = 1.2 to 0.9 points; (V) Reduced appetite = 1.2 to 0.2 points; (VI) Concentration difficulties = 3.3 to 2.1 points; (VII) Lassitude = 3.1 to 1.5 points; (VIII) Inability to feel = 3.0 to 1.1 points; (IX) Pessimistic thoughts = 1.8 to 0.9 points; (X) Suicidal thoughts = 0.8 to 0.0 points. Finally, all five patients with a MADRS score higher than 24 (markedly ill or extremely ill), presented at least a 50% reduction in MADRS score at week-12.

**Fig. 2:**
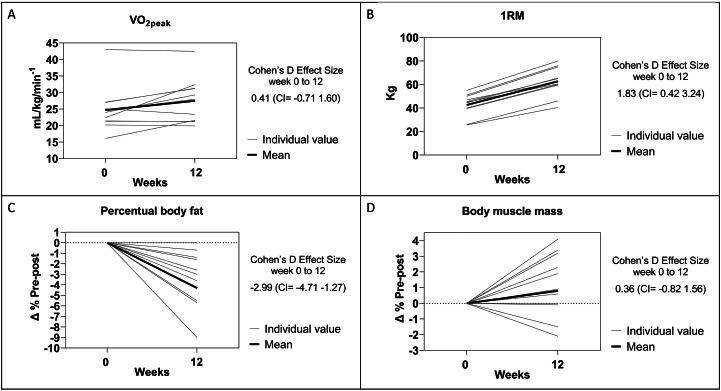
Results of cardiorespiratory fitness, muscular strength and body composition (Individual and mean value). Panel A = VO_2peak_ (Cardiorespiratory fitness); Panel B = 1RM (One repetition maximum - muscular strength); Panel C = Percentual of body fat; Panel D = Muscle mass. Change between baseline and 12 weeks and the Cohen’s d effect size

Finally, Figure 2 presents the VO_2peak_ (Panel A), muscular strength (Panel B), percentual body fat (Panel C), and body muscle mass (Panel D) considering baseline and 12 weeks. The Graphs show the individual values and mean of the weeks for tests (VO_2peak_, 1RM, percentual body fat and body muscle mass).

## Discussion

This pilot, open-label, single-arm study in patients diagnosed with BD demonstrated a robust improvement in depressive symptoms (MADRS) at 12 weeks of exercise (aerobic and resistance training) and 2, 4, 8, and 12 weeks compared to baseline, with a large and significant Cohen’s D effect. Five patients presented total remission (MADRS < 9 points) and four patients presented a reduction of more than 50% in depressive symptoms with a response rate of 82% (9/11). No significant changes were observed in manic symptoms, functioning, and quality of life. Additionally, all patients increased their muscular strength, and reduced the percentual body fat. Finally, six patients presented increased VO_2peak_, and eight patients increased their body muscle mass at week-12.

We highlight that the effect size detected in this pilot trial for the change in depressive symptoms (MADRS) is similar to the effect size (1.00, 95% CI -1.39 to -0.61) reported by Heisell et al., in a meta-analysis in MDD patients (Heissel et al. 2023). Additionally, studies with BD patients, such as the pilot study by Ng et al. (Ng et al. 2007), showed a reduction in depression scores using the self-reported 21-item version of the *Depression Anxiety Stress Scales* (DASS). Actually, this was the first study to investigate the effect of a walking group in BD. However, the study presented a retrospective design and only reported the benefits of a walking group, which is not considered a structured PE. Another study, by Sylvia et al., showed that 20 weeks of an intervention combining nutrition, exercise, and wellness in BD, promoted a change in depressive symptoms (MADRS) of four points (means of 17.2 to 13.2 points) and ES of 0.26 (non-significant) (Sylvia et al. 2019). However, an intervention for behavior change may not be comparable with PE, but a form of cognitive behavioral therapy to change behavior, and comparisons between our study and Sylvia’s study must be carried out with care. Finally, the study of Ashton et al. showed the association between PA and reduction in BD symptoms, but did not prescribe a PE intervention, but compared different PA routine levels in patients participating in a nutraceutical trial (Ashton et al. 2020). It is important to consider that PE interventions are usually programs with sessions of exercise (fixed frequency, duration, and orientation) and that the PA level is not a program of PE sessions per se. Instead, PA is the amount of movement involved in the routine (leisure, work, transport, etc.), usually carried out within a week. Even though our sample size was small, our study included patients with a MADRS score of 14 or more points (sample scores ranged from 14 to 40 points), indicating that our intervention reduced depressive symptoms in patients who were mildly, moderately, markedly and also extremely depressed.

For manic symptoms (YMRS), patients with scores greater than 12 were excluded from this study. In this sense, the group started the study with mild manic symptoms (mean at baseline = 3 points - outside the manic phase) and remained in this classification throughout the intervention period. As a result, there was no significant change in manic symptoms. However, compared to the Ng study which does not assess manic symptoms, our finding of no difference is positive evidence. Finally, our findings on manic symptoms contradict two cross-sectional studies linking exercise with manic episodes (Sylvia et al. 2013a; Wright et al. 2012).

For functioning (FAST), the high score presented here (baseline: mean = 39 points and week 12: mean = 37 points) showed our patients had moderate, and severe functional impairment, as categorized by the Bonnín et al. study (Bonnín et al. 2018). Despite the elevated score, no difference between baseline and 12 weeks was observed. As shown by a recent meta-analysis, even in the remission period, approximately 60% of patients with BD present a functional decrease when evaluated by the FAST (Léda-Rêgo and Bezerra-Filho 2020). To the best of our knowledge, no clinical trials have investigated the effect of exercise on the functioning score in BD. Other forms of intervention, as carried out during 21 weeks by Torrent et al. (Torrent et al. 2013) using functional remediation (therapy with neurocognitive techniques, training, psychoeducation on cognition-related issues, and problem-solving within an ecological framework) showed efficacy in improving the functional outcome of a sample of euthymic BD patients compared with treatment as usual. Considering the time difference between the intervention in Torrent’s study and our study, maybe a longer intervention of PE may result in a better impact on the FAST score.

Regarding the quality of life (WHOQOL), conflicting results may be highlighted in the subjects with psychiatric disorders. Schuch et al. showed that add-on PE for two weeks is an efficacious treatment for MDD patients, improving their quality of life (Schuch et al. 2015). It should be considered that there were severely depressed patients in the hospital, which is not similar to our sample. Considering schizophrenia patients, Heggelund et al. (Heggelund et al. 2012) showed no change in the quality of life after eight weeks of PE. In contrast to Schuch’s study, Heggelund’s study included also outpatients. We, therefore, speculate whether hospitalized patients with mental disorders may respond better to PE additional interventions for quality of life. Again, to our knowledge, no clinical studies have investigated the impact of exercise on the quality of life in BD.

Regarding the physical fitness variable, our study showed an increase in muscular strength and a decrease of percentual body fat of all patients. These finds agree with other studies that investigated the effect of aerobic plus strength training on muscular strength (Eklund et al. 2015; Pito et al. 2021) and percentual body fat (Sillanpää et al. 2008). Specially in BD patients, a decrease in percentual body fat is fundamental, since BD patients have increased overall and abdominal obesity, and they are considerably more likely to be obese than age- and gender-matched controls (Liu et al. 2022). Additionally, recent evidence suggests that obesity is an independent predictor of relapse and rehospitalization for severe mental illness (Manu et al. 2014). Even though no significant changes were verified in body muscle mass and VO_2peak_, more than 50% of patients increased these variables after the intervention, which can be impacted by the short duration of intervention.

Finally, our study has a high adherence (92% of sessions). The adherence to exercise programs has a big variance in studies that evolved physical exercise - adherence lower to 75% (Malmo et al. 2016) or bigger than 95% (Lucibello et al. 2020). Another side, our dropout is similar of summarized in the meta-analysis by Stubbs et al. (in unipolar depression), which was approximately 20% (Stubbs et al. 2016) and Vancampfort et al., (anxiety) at 22% dropout rate (Vancampfort et al. 2021). In resume, our study showed physical exercise program is acceptable and feasible in people with BD.

Our pilot study aimed to investigate the feasibility of an exercise protocol in subjects with bipolar depression. One important limitation was the small sample size and a lack of comparison group. We also did not include a longer follow-up period after the intervention, which limited our ability to measure the likelihood of eventual relapse and worsening of depressive symptoms after the intervention or our ability to detect long-term improvements in functioning and quality of life. The strengths of this study include the use of a structured and individual PE intervention, a validated method to confirm the diagnosis, and frequent measurements of depressive and manic symptoms during 12 weeks, which allowed us to detect no manic/mixed switches during the intervention.

Despite limitations, our study provides valid pilot data suggesting an adjuvant antidepressant effect of exercise in patients with BD, without triggering manic symptoms, and points to the importance of conducting randomized, controlled clinical trials to better confirm the promising effects of PE in BD.

## Data Availability

The datasets used and/or analyzed during the current study are available from the corresponding author upon reasonable request.

## List of abbreviations

BD: bipolar disorders
BMI: Body mass index
CI: confidence interval
CONSORT: Consolidated Standards of Reporting Trials
DASS: Depression Anxiety Stress Scales
ES: effect size
FAST: Functioning Assessment Short Test
HRmax: heart rate maximum
MADRS: Montgomery–Åsberg Depression Rating Scale
MDD: Major depressive disorder
Min: Minutes
One repetition maximal test: 1RM
PA: Physical activity
PE: Physical exercise
PROMAN: Bipolar Disorder Program
SCID: Structured Clinical Interview for DSM
SD: standard deviation
VO_2peak_: peak oxygen uptake
WHOQOL: World Health Organization Quality of Life
YMRS: Young Mania Rating Scale
